# Deletion of CD44 promotes adipogenesis by regulating PPARγ and cell cycle-related pathways

**DOI:** 10.1530/JOE-24-0079

**Published:** 2024-05-20

**Authors:** Xiong Weng, Hao Jiang, David J Walker, Houjiang Zhou, De Lin, Jing Wang, Li Kang

**Affiliations:** 1 Division of Cellular and Systems Medicine, School of Medicine, University of Dundee, Dundee, Scotland, UK; 2 Gene Expression and Regulation, School of Life Sciences, University of Dundee, Dundee, Scotland, UK; 3 MRC Protein Phosphorylation Unit, School of Life Sciences, Dundee, Scotland, UK; 4 Drug Discovery Unit, School of Life Sciences, University of Dundee, Dundee, Scotland, UK; 5 Science for Life Laboratory, Department of Biomedical and Clinical Sciences (BKV), Linköping University, Linköping, Sweden

**Keywords:** CD44, adipogenesis, insulin signalling, PPARγ, cell cycle

## Abstract

CD44, a cell surface adhesion receptor and stem cell biomarker, is recently implicated in chronic metabolic diseases. Ablation of CD44 ameliorates adipose tissue inflammation and insulin resistance in obesity. Here, we investigated cell type-specific CD44 expression in human and mouse adipose tissue and further studied how CD44 in preadipocytes regulates adipocyte function. Using Crispr Cas9-mdediated gene deletion and lentivirus-mediated gene re-expression, we discovered that deletion of CD44 promotes adipocyte differentiation and adipogenesis, whereas re-expression of CD44 abolishes this effect and decreases insulin responsiveness and adiponectin secretion in 3T3-L1 cells. Mechanistically, CD44 does so via suppressing *Pparg* expression. Using quantitative proteomics analysis, we further discovered that cell cycle-regulated pathways were mostly decreased by deletion of CD44. Indeed, re-expression of CD44 moderately restored expression of proteins involved in all phases of the cell cycle. These data were further supported by increased preadipocyte proliferation rates in CD44-deficient cells and re-expression of CD44 diminished this effect. Our data suggest that CD44 plays a crucial role in regulating adipogenesis and adipocyte function possibly through regulating PPARγ and cell cycle-related pathways. This study provides evidence for the first time that CD44 expressed in preadipocytes plays key roles in regulating adipocyte function outside immune cells where CD44 is primarily expressed. Therefore, targeting CD44 in (pre)adipocytes may provide therapeutic potential to treat obesity-associated metabolic complications.

## Introduction

Obesity is a metabolic complication of excess fat accumulation in organs, including adipose tissue, liver, skeletal muscle, and pancreas. The increasing obesity epidemic contributes to the prevalence of obesity-associated metabolic diseases, such as type 2 diabetes (T2D), non-alcoholic fatty liver disease, and cardiometabolic conditions (Stefan 2020, Klein *et al*. 2022). Over 670 million adults are obese worldwide, which causes huge socioeconomic burden to our society (NCD Risk Factor Collaboration, 2017). Despite great efforts being made into elucidating the pathogenesis of obesity, mechanisms of the development of obesity are not fully understood. Thus, it is important and urgent to identify novel therapeutic targets to advance the prevention and treatment of obesity and obesity-associated metabolic complications.

CD44 is a membrane protein and has different variants rising from alternative splicing in the *Cd44* gene (Ponta *et al*. 2003). The standard form of CD44 (CD44s) without alternative splicing variants is the smallest CD44 isoform (Zöller 2011). CD44s is ubiquitously expressed in a wide variety of tissues including the central nervous system, liver, lung, adipose tissue, and muscle (Weng *et al*. 2022). CD44 was extensively studied in cancer initiation and tumorigenesis, where increased expression of CD44 is often found as a biomarker of advanced tumour progression and poor prognosis in many cancers (Zöller 2011, Hassn Mesrati *et al*. 2021). However, recent studies suggest a potential role of CD44 in chronic metabolic diseases, such as obesity and T2D (Weng *et al*. 2022). Increased CD44 expression and deposition of its ligands, hyaluronan (HA) and osteopontin (OPN), were found in the adipose tissue of obese mice and humans (Kodama *et al*. 2012). The plasma CD44 level was positively correlated with insulin resistance and poor glycaemic control in human (Liu *et al*. 2015). Moreover, *Cd44* gene was implicated in the pathogenesis of T2D by a gene expression-based genome-wide association study (Kodama *et al*. 2012). Ablation of CD44 by genetic deletion or anti-CD44 monoclonal antibody administration reduced adipose tissue immune cell infiltration and insulin resistance in diet-induced obese mice (Kodama *et al*. 2015). These studies highlight that CD44 has critical roles in regulating adipose tissue function during obesity. Adipocytes are essential in maintaining adipose tissue function, and adipogenesis and insulin response are key characteristics of adipocyte function. However, how CD44 regulates adipogenesis and insulin signalling in adipocytes, therefore contributing to adipose tissue function is unclear.

In this study, we utilized CRISPR Cas9-mediated gene deletion and lentivirus-mediated gene re-expression in 3T3-L1 cells to determine the role of CD44 in adipogenesis and insulin response in adipocytes. Mechanistically, quantitative proteomics analysis was used to explore potential pathways regulated by CD44 in preadipocytes. We found that deletion of CD44 promoted adipogenesis and insulin response in 3T3-L1 cells, which were mediated by upregulation of *Pparg* and changes in cell cycle-related pathways.

## Materials and methods

### Mouse models

Male C57BL/6 mice were purchased from Charles River. Following 1-week acclimatization period, mice starting from the age of 7 weeks were fed a standard laboratory chow (13% calories as fat, LabDiet 5001) or a high fat diet (HFD) (60% calories as fat, SDS 824054) for 16 weeks to induce obesity. Animal experiments were carried out in compliance with the UK Animals (Scientific Procedures) Act 1986 and approved by the Animal Care and Use Committee of University of Dundee. All the mice were maintained in an air-conditioned room (22 ± 2°C) with a 12 h light:12-h darkness cycle and had free access to food and water.

### 3T3-L1 cell culture, differentiation, and insulin treatment

3T3-L1 cells were maintained in basal medium (4.5 g/L high-glucose DMEM, 10% Bovine Calf Serum, 1% Pen/Strep antibiotics, 2mM l-glutamine, and 5% 20 mM HEPES). Cells were differentiated in a medium containing 167 nM insulin, 0.5 mM 3-isobutyl-1-methylxanthine (IBMX), 1 μM dexamethasone (Dex), and 2 μM rosiglitazone (RAZ) for 4 days, followed by basal medium containing 167 nM insulin for another 3 days. Cells were then either collected for the measurement of adipogenesis (day 7 after differentiation initiation) or maintained in basal medium for another 7 days (Day 14 after differentiation initiation). For insulin treatment, cells were incubated with 100 nM insulin for 30 min.

### SGBS cell culture and differentiation

SGBS cells are a human preadipocyte cell line derived from patients with Simpson-Golabi Behmel syndrome, which provide a unique tool for studies of human adipocyte biology (Fischer-Posovszky *et al*. 2008). The SGBS cells were cultured in DMEM/F12 (#31330-38, Invitrogen) culture medium, containing 1% 3.3 mM biotin, 1% 1.7 mM panthotenate, 10% fatal serum, and 1% Pen/Strep antibiotics. SGBS cell differentiation was induced by the addition of 0.01 mg/mL transferrin, 20 nM insulin, 100 nM cortisol, 0.2 nM triiodothyronine (T3), 25 nM DEX, 250 μM IBMX, and 2 μM RAZ to the DMEM/F12 culture medium without serum for 4 days, followed by the exposure to the DMEM/F12 culture medium containing 0.01 mg/mL transferrin, 20 nM insulin, 100 nM cortisol, and 0.2 nM T3 with no serum for another 8–12 days.

### Oil Red O staining

Adipogenesis was assessed by Oil Red O staining. Cells were fixed in 4% formaldehyde for 2 h before being rinsed with 60% isopropanol and subsequently stained with Oil Red O solution (0.3% w/v) for 20 min at room temperature. Staining was quantified by measuring the absorbance at 540 nM.

### qRT-PCR

Total RNA was extracted by TRIzol reagent and synthesized into cDNA by SuperScript™ II Reverse Transcriptase kit (#18064014, ThermoFisher Scientific). qRT-PCR was run by either Taqman or SYBR^TM^ Green assays. The TaqMan probes for *Cd44* (Mm01277161_m1), *Pparg* (mm00440940_m1), *Cebpa* (Mm00514283_s1), and *18s* (Hs99999901_s1) were obtained from ThermoFisher. The primers of *Pref-1*: 5′-GGATTCGTCGACAAGACCTG-3′, 5′-GCTTGCACAGACACTCGAAG-3′; *Fabp4:* 5′-GATGCCTTTGTGGGAACCT-3′, 5′-CTGTCGTCTGCGGTGATTT-3′*; Scara5*: 5′-TGTGGAAGGTTCAGGATGCG-3′, 5′-GGCTTCGATTGCTTTCCACC-3′; and *18s*: 5′-GCAATTATTCCCCATGAACG-3′, 5′-GGCCTCACTAAACCATCCAA-3′ were obtained from Sigma. Data were normalized to 18 s and analysed using the 2^−ΔΔCt^ method.

### Western blot

1 × 10^6^ cells or ~50 mg mouse tissues were homogenized in protein lysis buffer (25 mM Tris–HCI pH 7.4, 50 mM NaF, 0.1 mM NaCI, 1 mM EDTA, 5 mM EGTA, 9.2% sucrose, 1% Triton X-100, 10 mM NaPp, 0.1% mercaptoethanol, 1 mM Na_3_VO_4_, 1 mM benzamidine, 0.1 M PMSF, and 10% glycerol). Protein concentration was quantified, and proteins were separated by the SDS-PAGE gel before being imaged by Western blotting. The primary antibody for CD44 (#A303-872A-M, BETHYL Laboratories), AKT (#9272, Cell Signaling), pAKT(S473) (#9212, Cell Signaling), adiponectin (#2789, Cell Signaling), and β-tubulin (#ab6046, Abcam) were used at 1:1000 dilution. The secondary antibody: anti-rabbit (#P/N:926-32213, LI-COR) and anti-sheep (#NL010, R&D) were used at 1:100,000 dilution.

### Crispr-Cas9 gene editing

Guide RNAs (gRNAs) targeting mouse CD44 (NM_001039151.1) were designed by Broad Institute Portal (https://singlecell.broadinstitute.org/single_cell). The top three gRNAs that target early exons with maximal on-target effects were selected and cloned into PX459 plasmids. Undifferentiated 3T3-L1 cells were transfected with lipofectamine LTX (#A12621; ThermoFisher Scientific) for 48 h, before being selected with 4 μg/mL puromycin. The Crispr-Cas9 editing efficiency was assessed by CD44 protein expression by Western. Cell populations with the least CD44 expression were sorted into single cells by fluorescence-activated cell sorting (FACS). The single-cell-derived stable CD44 knockout (KO) cell lines were further validated by CD44 protein expression and characterized by bi-allelic sequencing. For bi-allelic sequencing, genomic DNAs of CD44KO cell lines were isolated and DNA sequences around the gRNA targeted site were amplified by PCR using primers 5′-GTGGTAATTCCGAGGATTCA-3′ and 5′-GGCTGTTCATGGCTGTTC-3′. The PCR products were cloned into a pSC-AMP/Kan vector, using the StrataClone PCR Cloning Kit (#240205, Agilent). The bi-allelic sequencing was performed with the M13 reverse primer: 5′-GAGCGGATAACAATTTCACACAGG-3′. Cells that underwent Crispr-Cas9 editing but maintained normal CD44 protein expression were used as Crispr wildtype (CrisprWT) controls.

### Lentivirus-mediated CD44 re-expression

The CD44s DNA coding sequence (NP_001034240.1) was synthesized by Sigma and constructed into a lentivirus expressing vector *pLenti-CD44-C-mGFP-P2A-Puro* with a GFP tag. Both CD44 re-expressing vector (RE) and the control empty vector (EV) were packaged in HK293T cells, using the package plasmids PSPAX2 and PMD2.G with lipofectamine p3000 (#L3000075, ThermoFisher Scientific). CD44 re-expressing and control lentiviruses were then used to infect either CrisprWT or CD44KO 3T3-L1 cells for 48 h. Cells were then selected by 4 μg/mL puromycin for 72 h and the infection efficiency was validated by Western blot or qRT-PCR.

### Sample preparation for proteomics analysis

Undifferentiated CrisprWT EV, CD44KO EV, and CD44KO RE cells were prepared for proteomics analysis, following the single-pot solid-phase-enhanced sample preparation protocol (Hughes *et al*. 2019). Briefly, 1 × 10^6^ cells were lysed with 4% SDS in 100 mM TEAB buffer (#90114, ThermoFisher Scientific) and denatured at 95°C for 10 min. The cell lysates were then sonicated for 30 cycles (30 s on, 30 s off) before being treated with 10 mM DTT for 1 h for the reduction of reversibly oxidized cysteines. The proteins were then treated with 20 mM iodoacetamide for 45 min at room temperature to alkylate free thiols. The protein concentration was quantified and 100 µg proteins of each sample were incubated in acetonitrile with Sera-Mag SpeedBead Carboxylate-Modified Magnetic Particles (Hydrophilic) (#GE44152105050250, Merck) and Sera-Mag SpeedBead Carboxylate-Modified Magnetic Particles (Hydrophobic) (#GE24152105050350, Merck) for 10 min at room temperature. The beads were then washed and redissolved in 50 mM ammonium bicarbonate and digested overnight with trypsin at 37°C. After being acidified with 10% formic acid, the peptides were washed and eluted in 100 µL 2% DMSO. The samples were centrifuged at 10,000 ***g*** and the peptide containing supernatants were dried by speedvac. The peptides were redissolved in 100 mM TEAB buffer and equal amounts of peptides from each sample were labelled with TMT 10-plex Mass Tag labelling kit (#90110, ThermoFisher Scientific). After labelling, all samples were pooled and desalted before the mass spectrometry analysis.

### LC-MS and data analysis

MS analysis of TMT-labelled peptides was performed on a Q-exactive-HF mass spectrometer coupled with a Dionex Ultimate 3000RS (ThermoFisher Scientific). The detailed LC-MS protocol can be found in the supplemental materials. The LC-MS raw data were searched against the IPI mouse database version 3.83 using MaxQuant (1.6.6.0). The corrected reporter ion intensity of each protein intensity ratio was used for subsequent analysis; the protein intensity results of each group were processed by Perseus (2.0.6.0). All data were transformed into log2, regrouped according to genotypes and normalized to median. Unpaired Student’s *t*-test was performed to detect significantly changed proteins. Differentially expressed proteins (DEPs) were defined as proteins with a fold change >1.5 and a *P*-value <0.05. DEPs were visualized as volcano plots using Prism and the pathway enrichment was analysed by metascape.

### Single-cell sequencing data analysis

CD44 clustering expression in the subcutaneous and visceral white adipose tissue (WAT) of human and mice was analysed using published single-cell RNA sequencing data (SCP1376) at Single Cell Portal (https://singlecell.broadinstitute.org/single_cell). Briefly, 363,870 cells with 166,149 of human cells and 197 721 of mice were analysed according to their original clustering (Emont *et al*. 2022). The human single-cell data were integrated from paired visceral and subcutaneous WAT of plastic surgery biopsies of ten subjects and subcutaneous WAT from three adult males. The mouse WAT single-cell data contained gene expression profile of 197,721 cells, which integrated mouse subcutaneous and epididymal WAT of both sexes (10 males and 4 females) (Emont *et al*. 2022). For CD44 clustering expression in the epididymal WAT of obesity, single-cell RNA-seq data were derived from another public database (SCP1179) of male C57BL/6 mice fed either low fat diet (LFD) (Research Diets, 10% fat, D12450B) or HFD (Research Diets, 60% fat, D12492) (Sárvári *et al*. 2021). A 10X Genomics Chromium platform was used to sequence single-nuclear RNA-Sequence. A total of 19,723 cells were detected and clustered into seven different adipocyte subpopulations, including adipocytes (*n* = 4604), endothelial cells (*n* = 110), epididymal cells (*n* = 499), adipose stem and precursor cells (ASPCs) (*n* = 5171), immune cells (*n* = 8354), mesothelial cells (*n* = 680), and spermatozoa (n = 305). We extracted *Cd44* gene expression from each cell and performed differential expression analysis in different subpopulations using Seurat (Stuart *et al*. 2019).

### Cell proliferation assay

For real-time analysis of cellular proliferation, CrisprWT EV, CD44KO EV, and CD44KO RE cells were seeded in 96-well plates at a density of 1 × 10^3^ cells/well in 100 µL volume. Cells were seeded in the inner 60 wells, with the remaining 36 wells containing culture media. Cells were left for 15 min to settle and then placed in the IncuCyte S3 live cell imaging system (Sartorius, Surrey, UK). Each cell line was replicated in six wells per experiment. Four images per well were acquired every 2 h at a magnification of 10×, for a total of 8 days. Cell confluence was measured using a built-in AI algorithm using the IncuCyte S3 software (GUI Version 2022B Rev2).

### Statistical analysis

Real-time proliferation data was analysed using a generalized linear mixed-effects model using the ‘glmer’ function in the ‘lme4’ package in R (version 3.3), followed by an ANOVA using the ‘car’ package to obtain a corrected *P*-value. The data were analysed this way due to non-normality of model residuals and to accommodate the repeated measures element (timepoint and biological replicate). All other data were analysed by either unpaired Student’s *t*-test or one-way or two-way ANOVA for statistical significance as indicated. *Post* *hoc* pairwise comparisons were performed using Tukey’s method. Data were presented as mean ± s.e.m. and the significant level was ^*^P < 0.05, ^**^P < 0.01, ^***^P < 0.005, and ^****^P < 0.001. All data figures were generated by Prism (GraphPad).

## Results

### CD44 expression was regulated by obesity and during adipocyte differentiation

**Figure 1:**
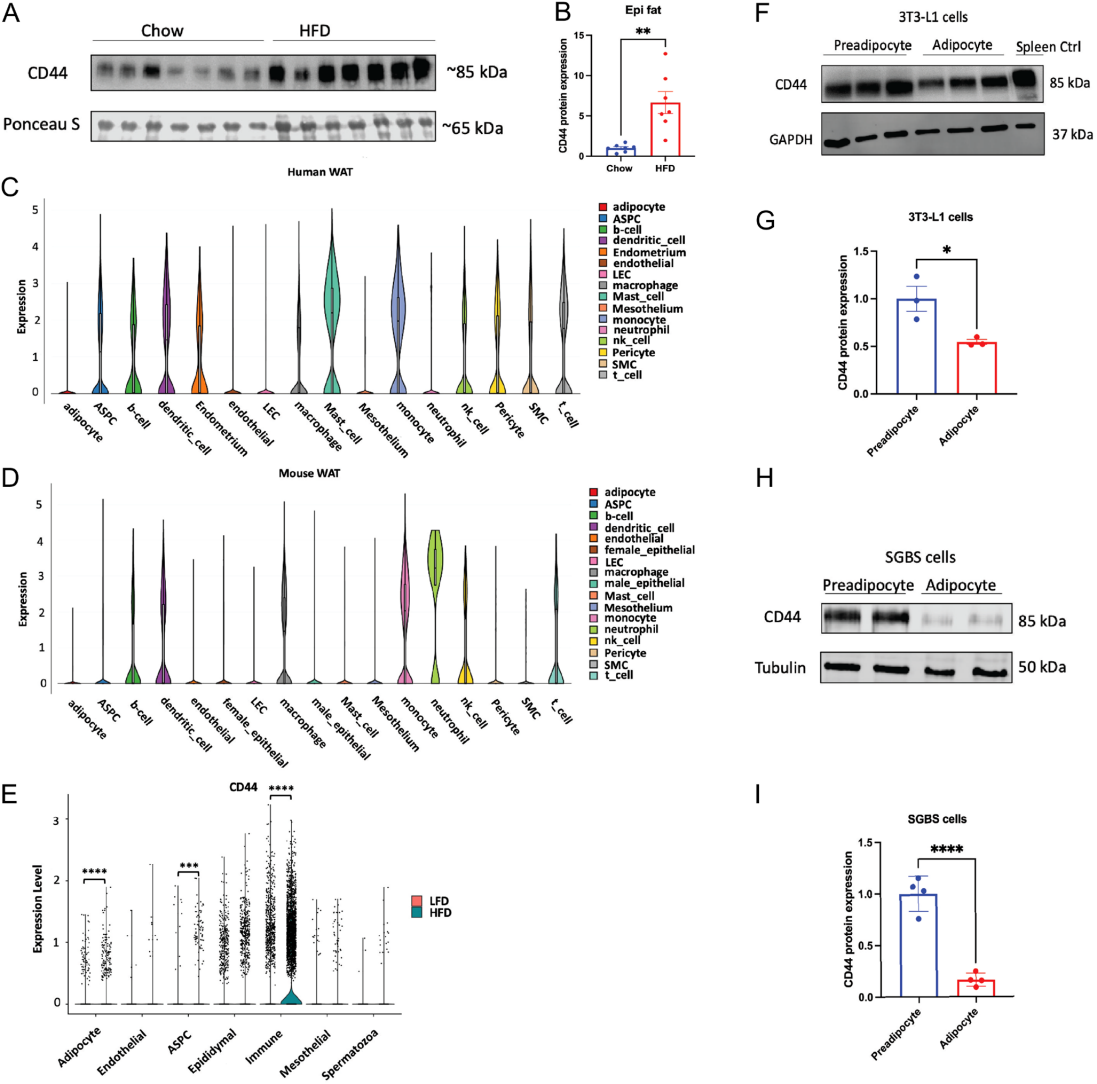
CD44 expression in adipose tissue, preadipocytes, and adipocytes. (A and B) C57BL/6 mice were fed with either a chow diet (D/811004; DBM) or a 60% high-fat diet (HFD) (#824054; SDS) for 16 weeks. CD44 protein expression was determined in the epididymal WAT by Western blot. (C and D) CD44 clustering expression in the subcutaneous and visceral WAT of human and mice was analysed using published single-cell RNA sequencing data (SCP1376) at Single Cell Portal (https://singlecell.broadinstitute.org/single_cell) (Emont *et al.* 2022). (E) CD44 expression in different cell clusters in the epididymal WAT of male C57BL/6 mice fed 16 weeks of low-fat diet (LFD, 10% fat) or HFD diet (60% fat) was analysed using published single-cell RNAseq data (SCP1179, https://singlecell.broadinstitute.org/single_cell)(Sárvári *et al.* 2021). (F and G) CD44 protein expression and quantification in 3T3-L1 preadipocytes and 14-day differentiated adipocytes. (H and I) CD44 protein expression and quantification in SGBS preadipocytes and 16-day differentiated adipocytes. Unpaired Student’s *t*-test was performed for statistical comparison. *P < 0.05; **P < 0.01; ***P < 0.005; ****P < 0.0001.

CD44 protein was increased five-fold in the epididymal WAT of HFD-fed male mice compared to those of chow-fed males (Fig. 1A and B). The expression pattern of CD44 in mouse and human WAT was next analysed using published single-cell sequencing data (Emont *et al*. 2022). It was shown that CD44 was ubiquitously expressed with highest expression in mast cells and monocytes in human WAT but in neutrophils and monocytes in mouse WAT (Fig. 1C and D). Despite relatively low expression levels, CD44 was expressed in ASPCs (Fig. 1C and D), which give rise to mature adipocytes and are essential for maintaining adipose tissue function. Furthermore, we compared the expression level of CD44 in epididymal WAT of mice fed with LFD (10% fat) or HFD (60% fat) using another single-cell sequencing dataset (Sárvári *et al*. 2021). It was found that HFD feeding in mice increased the numbers of CD44 expressing cells, especially CD44 expressing immune cells, ASPCs, and adipocytes (Fig. 1E).

In 3T3-L1 cells, protein expression of CD44 was decreased after adipocyte differentiation (Fig. 1F and G). Likewise, CD44 protein expression was downregulated in human SGBS adipocytes after differentiation (Fig. 1H and I). These data suggest that CD44 was regulated in adipose tissue during obesity and expressed in preadipocytes, whose expression level was downregulated during adipocyte differentiation, suggesting a potential role of CD44 in regulating adipogenesis contributing to the pathogenesis of obesity.

### Deletion of CD44 in preadipocytes promotes adipogenesis

**Figure 2:**
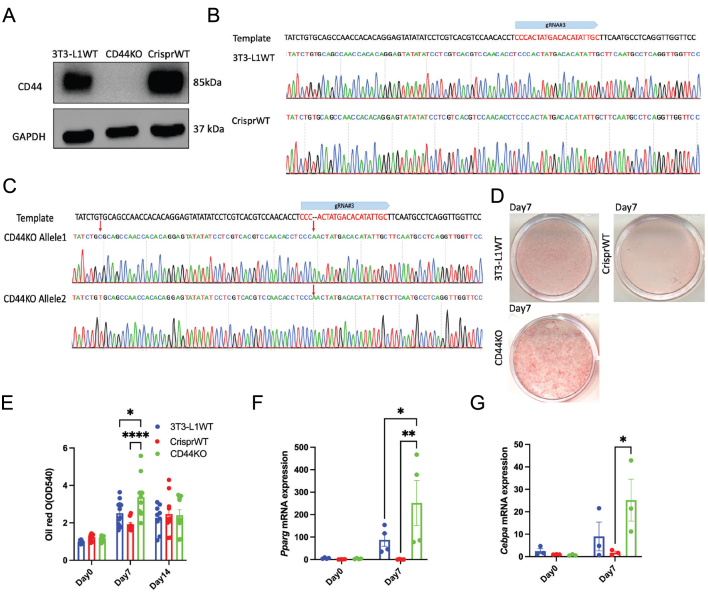
Deletion of CD44 promoted adipogenesis in 3T3-L1 cells. (A) Representative CD44 expression in 3T3-L1 naïve cells (3T3-L1WT), Crispr WT, and CD44KO cells. (B) Bi-allelic sequencing of 3T3-L1WT and Crispr WT cells. (C) Bi-allelic sequencing results of CD44KO cells. (D and E) Representative images of Oil Red O staining and quantification during the differentiation of 3T3-L1 WT, Crispr WT, and CD44KO cells. (F and G) *Pparg* and *Cebpa* mRNA expression on day 0, and day 7 after differentiation in 3T3-L1 WT, Crispr WT, and CD44KO cells. Two-way ANOVA with Tukey's multiple comparisons was performed to analyse the data. *P < 0.05; **P < 0.01; ***P < 0.005; ****P < 0.0001.

To further study how CD44 regulates adipogenesis, we generated stable, CD44KO 3T3-L1 cells by Crispr Cas9-mediated gene editing. Three gRNAs targeting different exons of murine *Cd44* gene were designed (Supplementary Figure 1A) and constructed into Crispr Cas9 expressing vectors (PX459). The constructions were confirmed by enzymatic digestion and DNA sequencing (Supplementary Figure 1B and C). The 3T3-L1 preadipocytes were transfected by individual gRNA. The gRNA#3-transfected cells had the most decreased CD44 expression when compared to other gRNAs and vector alone transfected cells (Supplementary Figure 1D) and therefore were further sorted into single-cell colonies. The stable CD44KO single-cell lines were then screened for complete CD44 protein ablation (Fig. 2A). Cells that underwent Crispr Cas9 editing but maintained normal CD44 protein expression were used as CrisprWT controls (Fig. 2A). To further characterise the CD44KO cells, DNA sequences around the gRNA#3 targeted site were amplified from each single-cell line and bi-allelically sequenced. 3T3-L1 naive WT cells and the CrisprWT cells had identical DNA sequence in both alleles of the *Cd44* gene (Fig. 2B). However, CD44KO cells had one base of A inserted in both alleles and a T-to-C mutation in the upstream of the gRNA#3 targeted site in one of the alleles (Fig. 2C).

To determine how CD44 regulates adipogenesis, naïve 3T3-L1WT, CrisprWT, and CD44KO cells were differentiated for 14 days. Adipogenesis was assessed by Oil Red O staining. The adipogenic induction significantly increased Oil Red O staining after 7 and 14 days of differentiation in all cells (Fig. 2D and E). Interestingly, CD44KO cells had significantly higher Oil Red O staining when compared to both 3T3-L1WT and CrisprWT cells at day 7 but not at day 14 (Fig. 2D and E). These results suggest that deletion of CD44 promoted early adipogenesis in 3T3-L1 cells. In agreement, mRNA levels of *Pparg* and *Cebpa* were significantly increased in CD44KO cells on day 7 when compared to CrisprWT cells (Fig. 2F and G).

### Lentivirus-mediated re-expression of CD44 attenuated adipogenesis in the CD44KO cells by suppressing *Pparg*

**Figure 3:**
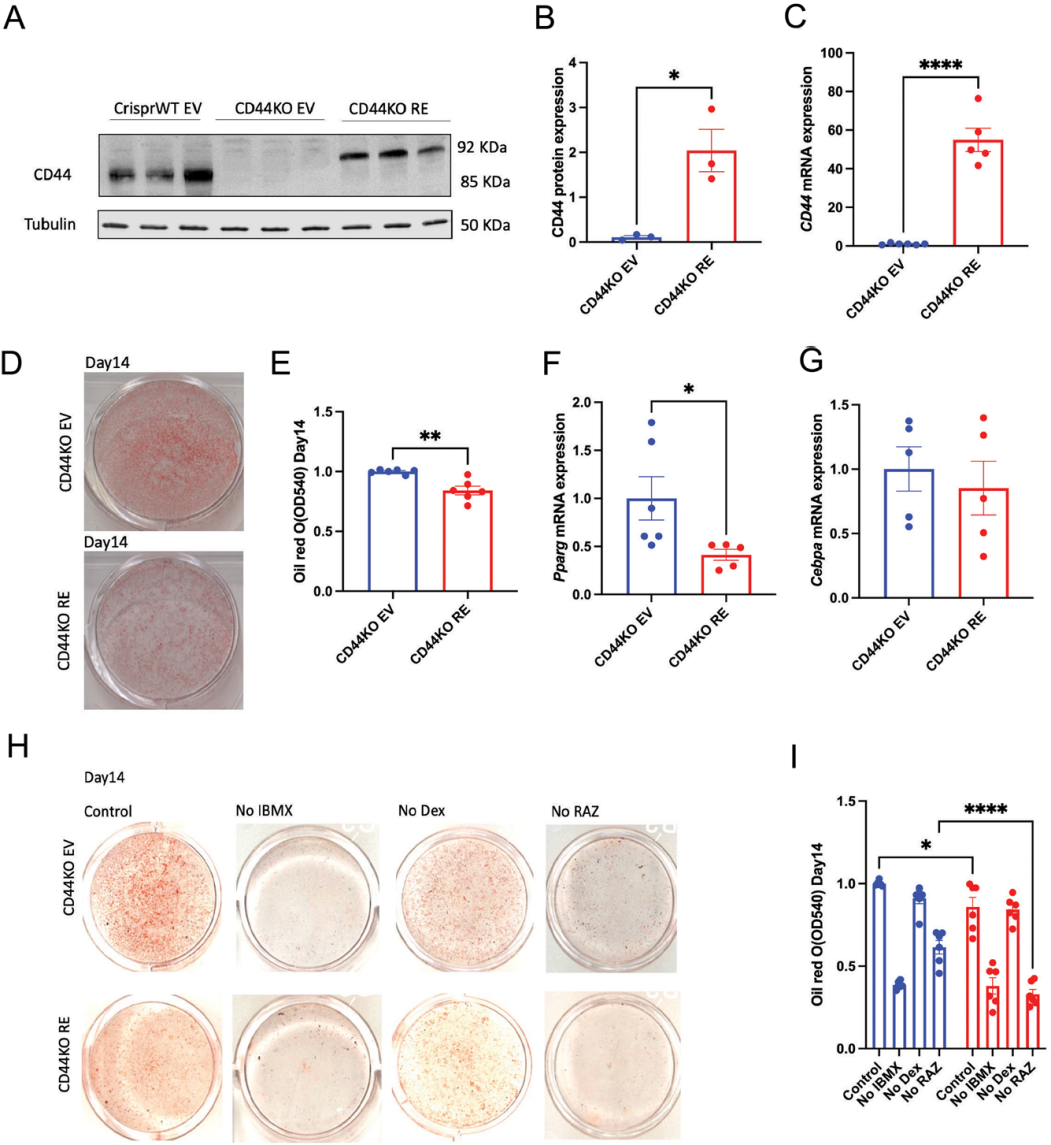
CD44 re-expression attenuated adipogenesis via suppressing *Pparg* expression. (A) Representative Western blot of CD44 expression. (B) Quantification of CD44 protein expression. (C) mRNA expression of CD44 in CD44KO EV (control empty vector) and CD44KO cells re-expressing CD44 (CD44KO RE). (D and E) Representative images of Oil Red O staining and quantification on day 14 of adipocytes differentiation in CD44KO EV and CD44KO RE cells. (F and G) *Pparg* and *Cebpa* mRNA expression in CD44KO EV and CD44KO RE cells after 14 days of differentiation. (H) CD44KO EV and CD44KO RE cells were differentiated for 14 days in adipogenic induction medium with/without specific agonists. IBMX, 3-isobutyl-1-methylxanthine; Dex, dexamethasone; RAZ, rosiglitazone. Unpaired Student’s *t*-test or two-way ANOVA with Tukey’s multiple comparisons was performed for statistical analysis. *P < 0.05; **P < 0.01; ***P < 0.005; ****P < 0.0001.

We next investigated CD44-specific effects on adipogenesis by re-expressing CD44 in the CD44KO cells. The expected molecular weight of the recombinant CD44 protein is 92KDa, which was present in CD44KO cells re-expressing CD44 (CD44KO RE) but not in the empty vector transfected CD44KO cells (CD44KO EV) (Fig. 3A and B). In agreement, CD44 mRNA levels were also increased in CD44KO RE cells (Fig. 3C). We next measured adipogenesis in these cells. CD44 re-expression caused a 20% reduction in Oil Red O staining on day 14 in CD44KO cells when compared to CD44KO EV cells (Fig. 3D and E), accompanied by decreased *Pparg* mRNA levels (Fig. 3F). However, the mRNA expression of *Cebpa* was not changed between CD44KO RE and CD44KO EV cells (Fig. 3G). These data suggest that CD44 decreases *Pparg* expression, therefore inhibiting adipogenesis.

To further confirm these results, we conditionally removed the agonists of various adipogenic stimuli in the differentiation medium of CD44KO EV and CD44KO RE cells. The cells were then differentiated for 14 days and adipogenesis was assessed. Consistently, CD44 re-expression significantly decreased Oil Red O staining in CD44KO cells when incubated with control differentiation cocktail (Fig. 3H and I). Removal of IBMX, an activator of cAMP-associated pathway, significantly attenuated adipogenesis in both CD44KO EV and CD44KO RE cells, suggesting that activation of cAMP-associated signals was required for adipogenesis independent of CD44 expression. In contrast, removal of Dex, an activator of glucocorticoid receptor-associated pathway, had no significant effects on adipogenesis, indicating that CD44-mediated adipogenic regulation was unlikely through glucocorticoid receptor-associated pathways. Interestingly, the adipogenic cocktail without RAZ, a *Pparg* agonist attenuated adipogenesis in both CD44KO EV and CD44KO RE cells, when compared to the control cocktail, but to a much greater extend in the CD44KO RE cells when compared to CD44KO EV cells (Fig. 3H and I). These results further demonstrate that CD44 inhibits adipogenesis by suppressing *Pparg* expression.

### CD44 re-expression in CD44KO cells impaired adipocyte function by decreasing insulin signalling and adiponectin secretion

**Figure 4:**
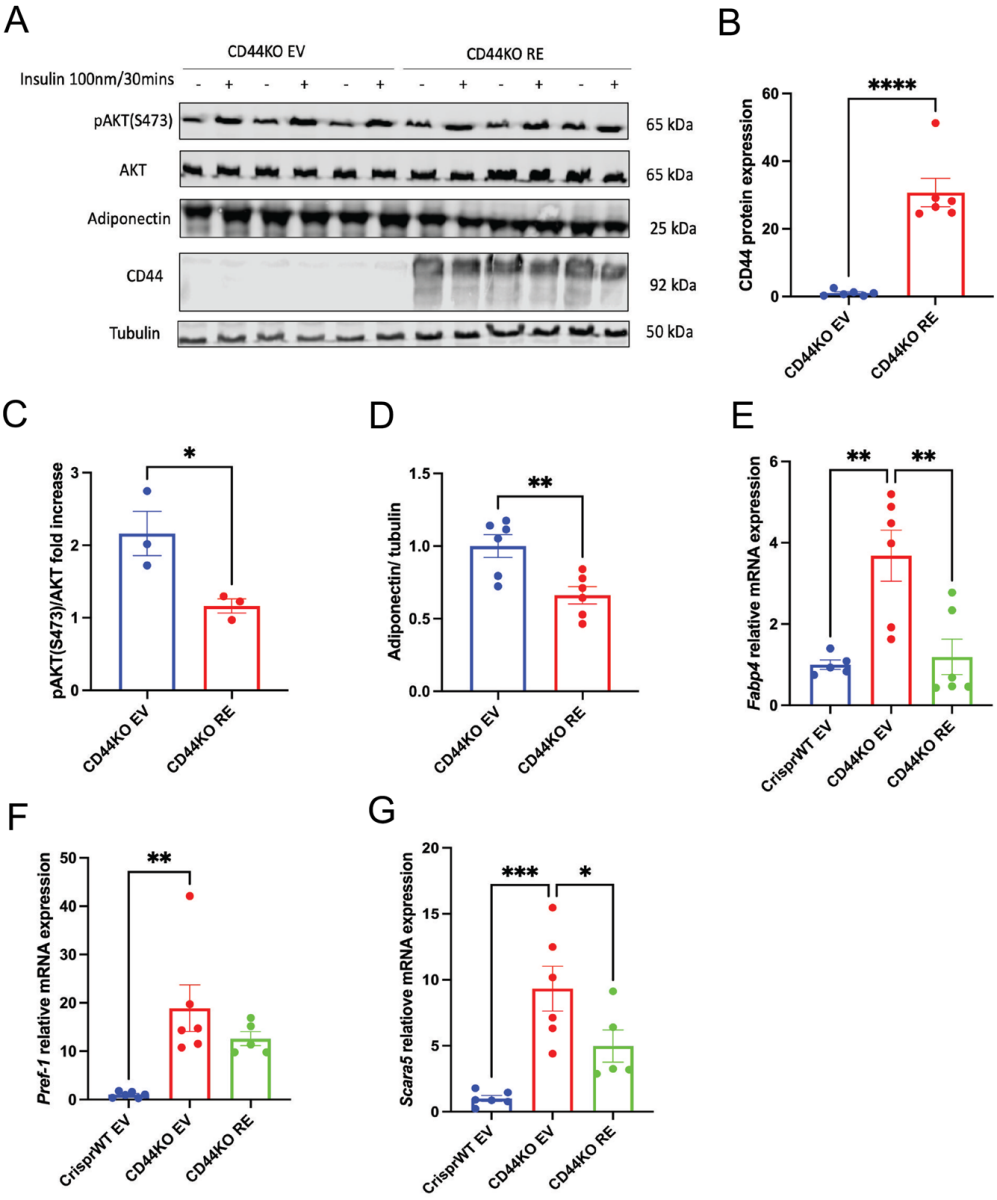
CD44 re-expression decreased insulin responsiveness and adiponectin secretion. (A) CD44KO EV and CD44KO RE cells were differentiated for 14 days. Cells were treated with or without insulin at 100 nM for 30 min. Cell lysates were prepared for the measurements of pAKT(S473), AKT, adiponectin, and CD44 by Western blot. (B−D) Quantification of CD44 protein expression, fold increase of insulin stimulated pAKT(S473) phosphorylation, and adiponectin in CD44KO EV and CD44KO RE cells. CD44 and adiponectin levels were averages of both insulin-stimulated and non-insulin-stimulated states. (E−G) *Fabp4, Pref-1* and *Scara5* mRNA expression in CrisprWT EV, CD44KO EV, and CD44KO RE preadipocytes. Unpaired Student’s *t*-test or one-way ANOVA with Dunnett's multiple comparisons were used for statistical analysis. *P < 0.05; **P < 0.01; ***P < 0.005; ****P < 0.0001.

To examine whether CD44 re-expression attenuates adipocyte function, CD44KO EV and CD44KO RE cells were treated with 100 nM insulin for 30 min after 14 days of differentiation. CD44 protein expression was sustained in CD44KO RE cells post differentiation (Fig. 4A and B). The insulin response was decreased in CD44KO RE cells, evidenced by decreased insulin-stimulated AKT phosphorylation (S473) when compared to CD44KO EV cells (Fig. 4A and C). In addition, adiponectin, a metabolically favourable hormone secreted by adipocytes, was decreased in differentiated CD44KO RE cells relative to CD44KO EV cells (Fig. 4A and D), suggesting an impaired secretory/endocrine function of adipocytes.

To further characterise the CD44-deficient and re-expressing cells, mRNA levels of additional adipogenic markers and regulators were measured. *Fabp4* is a fatty acid-binding protein and identified as a marker of terminally differentiated adipocytes (Cao *et al*. 2013). *Fabp4* mRNA expression was significantly increased in CD44KO EV cells relative to CrisprWT EV cells but was restored in CD44KO RE cells (Fig. 4E). *Pref-1* is a preadipocyte growth factor (Moon *et al*. 2002, Nueda *et al*. 2007), and its mRNA expression was increased 20-fold in CD44KO EV when compared to CrisprWT EV cells (Fig. 4F). However, there was no significant difference in *Pref-1* mRNA between CD44KO EV and CD44KO RE cells (Fig. 4F). *Scara5* is a scavenger receptor class A member protein, and previous study suggested that knockdown of *Scara5* inhibited adipogenesis in C3H10T1/2 pluripotent stem cells and A33 cells (Lee *et al*. 2017). Here, we found that deletion of CD44 significantly increased mRNA expression of *Scara5*, while CD44 re-expression attenuated its mRNA expression (Fig. 4G). Taken together, these data further characterized the enhanced adipogenic capacity of CD44KO cells and re-expression of CD44 abolished this augmentation, suggesting a CD44-specific role in regulating adipogenesis.

### A distinct clone of CD44-deficient cells exhibited consistent phenotype with enhanced adipogenesis and re-expression of CD44 in these cells impaired adipogenesis and adipocyte function

**Figure 5:**
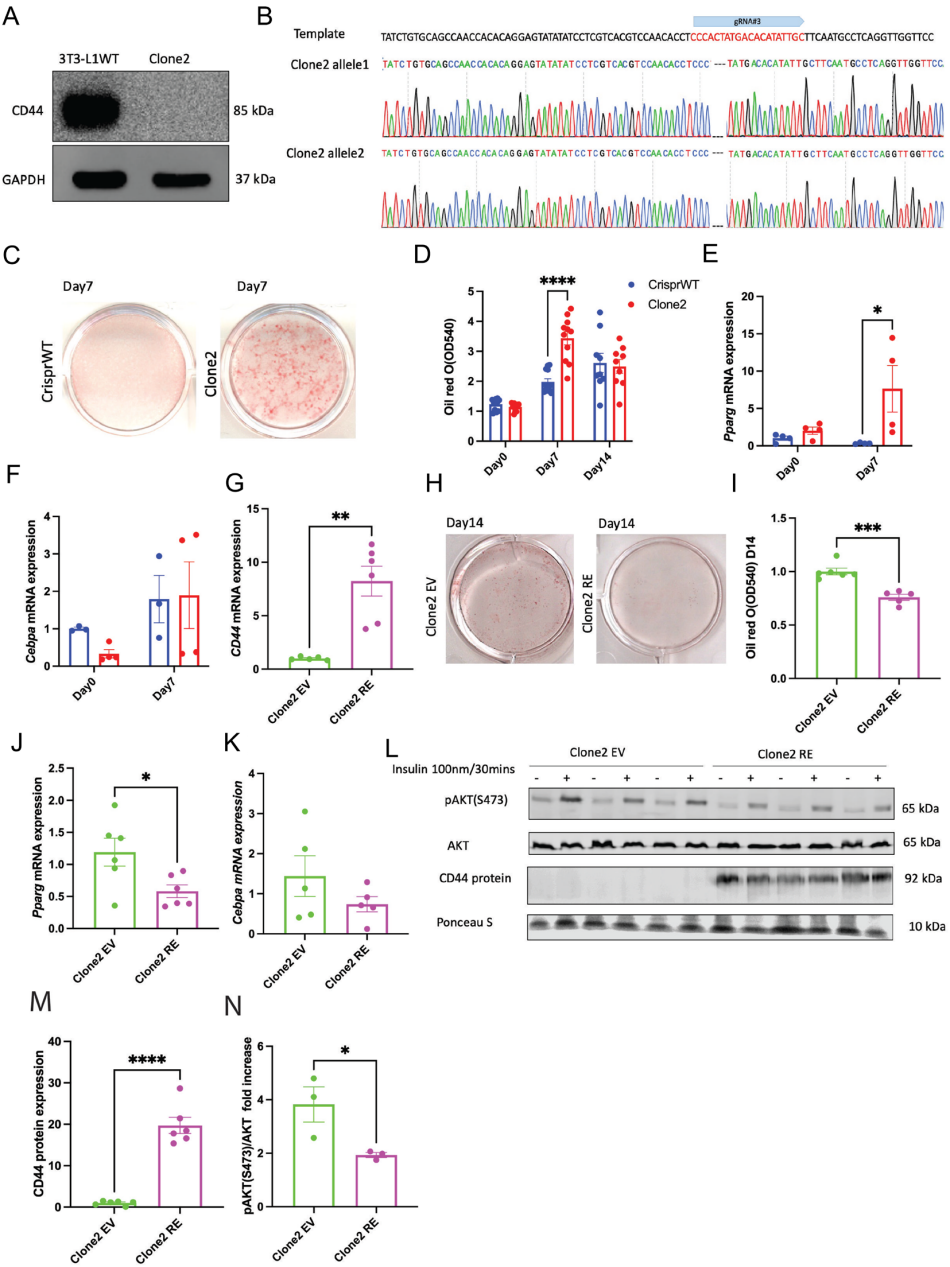
A distinct clone of CD44-deficient cells (Clone2) exhibited consistent phenotypes with CD44KO cells. (A) Representative CD44 expression in 3T3-L1 naïve cells (3T3-L1WT) and Clone2 cells. (B) Bi-allelic sequencing results of Clone2 cells. (C and D) Representative images of Oil Red O staining and quantification during the differentiation of CrisprWT and Clone2 cells. (E and F) *Pparg* and *Cebpa* mRNA expression on day 0, and day 7 after differentiation in CrisprWT and Clone2 cells. (G) mRNA expression of CD44 in Clone2 EV and Clone2 RE cells. (H and I) Representative images of Oil Red O staining and quantification on day 14 of adipocyte differentiation in Clone2 EV and Clone2 RE cells. (J and K) *Pparg* and *Cebpa* mRNA expression in Clone2 EV and Clone2 RE cells after 14 days of differentiation. (L) Representative Western blots of pAKT(S473), AKT and CD44 in Clone2 EV and Clone2 RE cells after 14 days of differentiation with or without insulin stimulation (100 nM for 30 min). (M and N) Quantification of CD44 protein expression and fold increase of insulin stimulated AKT(S473) phosphorylation in Clone2 EV and Clone2 RE cells. Unpaired Student’s *t*-test or two-way ANOVA with Tukey's multiple comparisons were used for statistical analysis. *P < 0.05; **P < 0.01; ***P < 0.005; ****P < 0.0001.

To avoid potential biases in our findings, we selected another clone of CD44-deficient cells (Clone2) with distinct gene editing. CD44 protein was absent in Clone2 cells (Fig. 5A). Clone2 cells had two bases of AC deleted in both alleles (Fig. 5B). Consistent with the phenotype of the other CD44KO clone, Clone2 cells had significantly higher Oil Red O staining 7 days after differentiation relative to CrisprWT cells (Fig. 5C and D). Increased Oil Red O staining was accompanied by increased *Pparg* mRNAs but not *Cebpa* mRNAs on day 7 (Fig. 5E and F). Re-expression of CD44 in Clone2 cells resulted in an increased *cd44* gene expression (Fig. 5G). Furthermore, re-expression of CD44 in Clone2 cells led to a reduction in Oil Red O staining 14 days after differentiation (Fig. 5H and I), which was accompanied with decreased *Pparg* mRNAs (Fig. 5J). *Cebpa* mRNAs, however, was not significantly affected by CD44 re-expression in Clone2 cells (Fig. 5K). Moreover, consistent with findings in the other CD44KO clone, re-expression of CD44 in Clone2 cells displayed sustained CD44 protein expression and decreased insulin-stimulated phosphorylation of AKT (S473) after 14 days of differentiation (Fig. 5L, M, and N).

### Proteomics analysis of CrisprWT EV, CD44KO EV, and CD44KO RE cells

**Figure 6:**
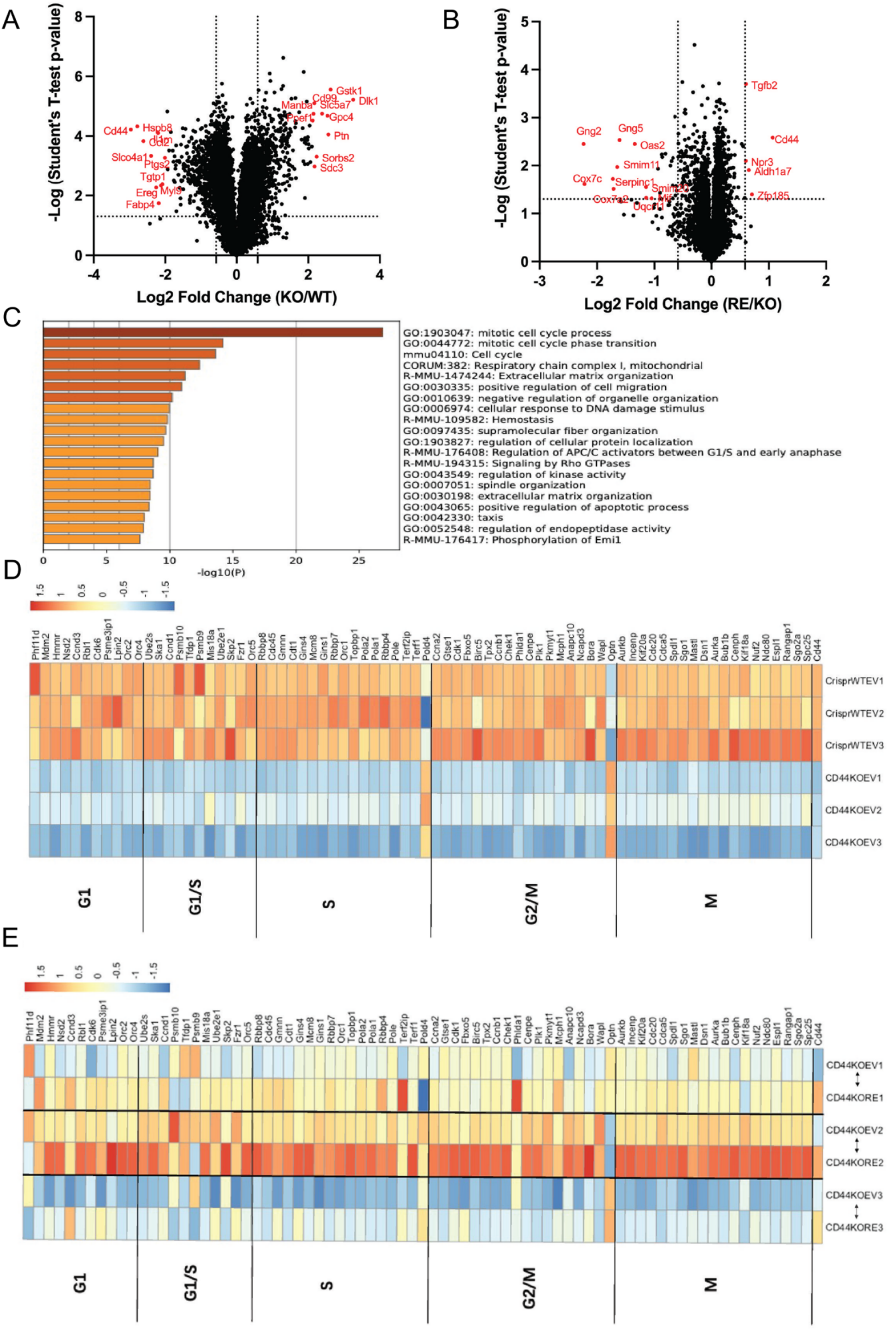
CD44 regulated adipogenesis possibly through cell cycle-related proteins. (A) Volcano plot showing differential protein expression in CD44KO EV vs CrisprWT EV cells. Data were plotted by log2 fold change (KO/WT) to −log (Student’s *t*-test *P-*value). Top ten up- and down-regulated differentially regulated proteins (DEPs) were marked in red. (B) Volcano plot showing differential protein expression in CD44KO RE vs CD44KO EV cells. Top ten downregulated DEPs and five upregulated DEPs were marked in red. (C) Pathway enrichment analysis between CrisprWT EV vs CD44KO EV cells by metascape. (D and E) Heatmaps representing cell cycle-related protein expression in CrisprWT EV vs CD44KO EV and CD44KO RE vs CD44KO EV with KEGG pathway enrichment cluster go:1903047, go:0044772, and mmu04110. Heatmaps were generated by pheatmap package in R (version 4.2.3).

To further explore potential mechanisms of how CD44 regulates adipogenesis and adipocyte function, we employed proteomics analysis in CrisprWT EV, CD44KO EV, and CD44KO RE preadipocytes. Proteomics data were processed using Perseus (Supplementary Figure 1E). In total, 8541 proteins were identified from all three cell lines. A subset of 1087 DEPs were observed while comparing CD44KO EV to CrisprWT EV cells, with 548 proteins decreased and 539 proteins increased in the CD44KO EV cells. The proteomics results were visualized in volcano plots (Fig. 6A), where the top ten most-changed DEPs were marked. Surprisingly, between CD44KO RE and CD44KO EV cells only 30 DEPs were identified (Fig. 6B).

The pathway enrichment was further analysed using identified DEPs by metascape. Cell cycle-related pathways, such as mitotic cell cycle process, mitotic cell cycle phase transition, and cell cycle, were mostly enriched by CD44 deletion (Fig. 6C). This implied that CD44 might regulate adipogenesis through regulating cell cycle progression. However, the pathway enrichment analysis between CD44KO RE and CD44KO EV cells was not possible due to small numbers of DEPs identified. Next, we analysed the protein expression of cell cycle related genes in the proteomics dataset. Deletion of CD44 caused a dramatic downregulation in proteins involved in all phases of cell cycle except for Pold4 (DNA polymerase delta 4) and Optn (optineurin) (Fig. 6D). When CD44 was re-expressed in each of the three biological repeats (CD44KO RE vs CD44KO EV), the downregulation of most cell cycle-related proteins was rescued (Fig. 6E).

### Deletion of CD44 in preadipocytes promotes cell proliferation

**Figure 7:**
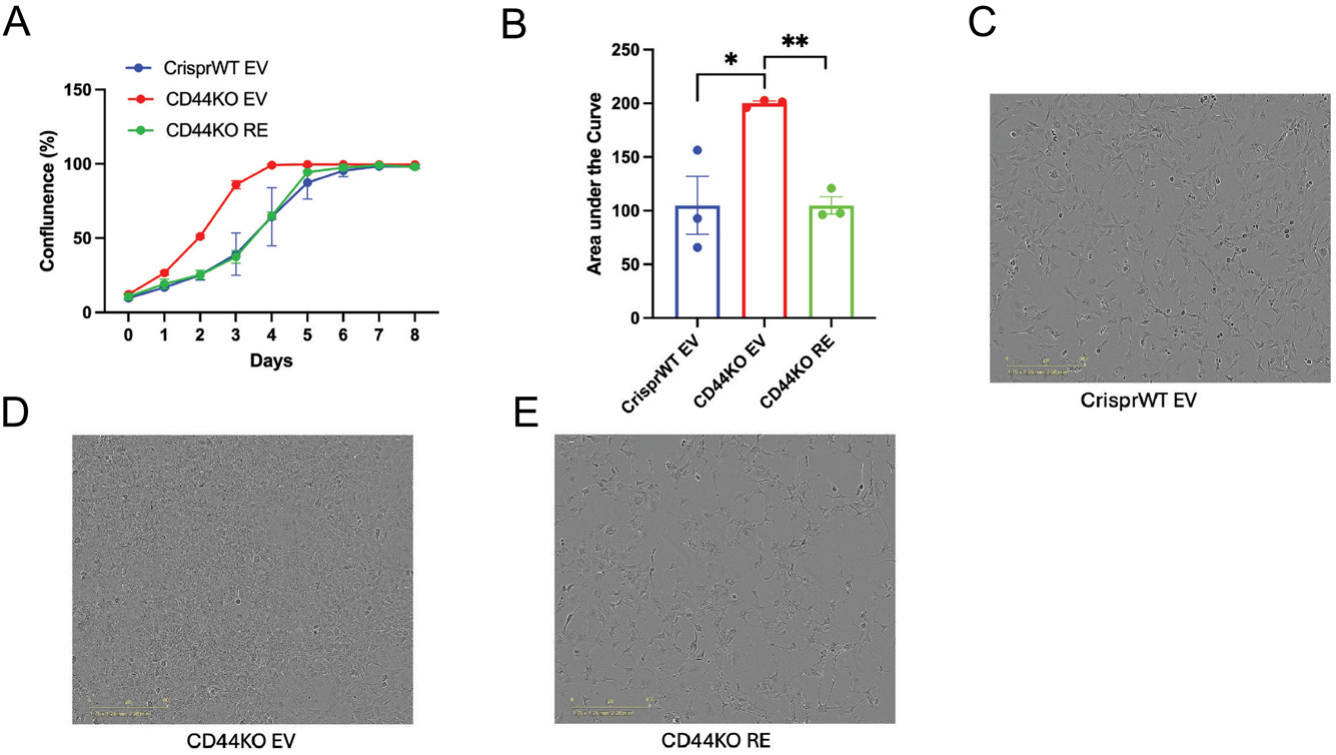
Deletion of CD44 in preadipocytes promoted cell proliferation. (A) Growth curves of cell proliferation assay of CrisprWT EV, CD44KO EV, and CD44KO RE cells for 8 days. (B) Areas under the curves (AUCs) for days 0–4 of the proliferation assay curves. (C–E) Representative images of CrisprWT EV, CD44KO EV, and CD44KO RE cells on day 4 of the proliferation assay. Data are means ± s.e.m. from three separate experiments. Real-time proliferation data were analysed using a generalized linear mixed-effects model using the ‘glmer’ function in the ‘lme4’ package in R (version 3.3), followed by an ANOVA using the ‘car’ package to obtain a corrected *P*-value. *P < 0.05; **P < 0.01.

As suggested by proteomics data, CD44 may regulate adipogenesis via altering cell cycle progression. Therefore, we performed cell proliferation assay. CD44KO EV cells proliferated at a much faster rate compared to CrisprWT EV cells (Fig. 7). This effect was restored when CD44 was re-expressed in the CD44KO cells, as evidenced by the cell growth curve (Fig. 7A), areas under the curves between 0 and 4 days (Fig. 7B), and microscopic images (Fig. 7C, D, and E).

## Discussion

CD44, a well-known stem cell biomarker, is recently implicated in chronic metabolic diseases (Weng *et al*. 2022). CD44 and its ligands HA and OPN were increased in the adipose tissue of obese mice (Nomiyama *et al*. 2007, Kiefer *et al*. 2008, Kang *et al*. 2013, Zhu *et al*. 2021, Romo *et al*. 2022). Here, we found for the first time that CD44 expression in preadipocytes hinders adipocyte differentiation and adipogenesis, therefore negatively impacting adipocyte function including insulin responsiveness and its secretory capacity. Mechanistically, CD44 does so possibly through suppressing adipogenic transcription factor *Pparg* expression and cell cycle-related pathways.

CD44 has previously shown to be primarily expressed in inflammatory cells such as macrophages in obese adipose tissue and CD44 expression positively correlated with inflammatory markers CD68 and IL6 gene expression in the subcutaneous WAT of obese human (Kodama *et al*. 2012, Liu *et al*. 2015). In consistent with these findings, we observed that CD44 was highly expressed in mast cells and monocytes in human WAT, and monocytes and neutrophils in mouse WAT. But we observed that CD44 was also expressed in ASPCs in human WAT. Moreover, in obese mice, in addition to immune cells, CD44-expressing cells increased, especially ASPC cells and adipocytes. These results support the notion that CD44 may be important in regulating adipose function through its direct action in adipocytes or adipocyte precursor cells.

Adipose* Cd44* gene was associated with T2D by an expression-based-genome wide association study (Kodama *et al*. 2012); however, it is unclear which mutations in the coding region of *Cd44* or which CD44 variant may be involved. Using CRISPR Cas9-mediated gene editing technique, we established two stable CD44KO cell lines (CD44KO cells and Clone2 cells), which adopted distinct genetic decorations to ablate CD44. Interestingly, despite the distinct genetic manipulations, both KO cells exhibited same phenotypes including enhanced adipogenesis and increased *Pparg* mRNA expression, which could be rescued by re-expression of CD44. Stably cloned cell lines using CRISPR Cas9 editing technique possess both advantages (e.g. higher gene-editing efficiency and no DNA integration) (Yang & Yang 2021) and disadvantages (e.g. lack of on-target editing efficiency (Peng *et al*. 2016). In addition, while the gRNAs guide the Cas9 protein to break DNA sequences of interest in the genome, it can also promote Cas9 protein to mismatch targeted DNA sequences (Zhang *et al*. 2015), resulting in off-target effects. However, if any, these off-target effects will be equivalent between the CrisprWT control cells and the two KO cell lines. In comparison to stably cloned cell lines, acute gene knockdown approach using siRNA was also considered, yet this approach caused a dynamic and differential regulation of CD44 expression during adipogenesis (Supplementary Figure 1F). Therefore, stably cloned CD44 KO cells were used to address how complete deletion of CD44 affects adipogenesis and adipocyte function in the current study.

Here, we observe an inhibitory role of CD44 in adipogenesis, at least in 3T3-L1 cells, as deletion of CD44 promoted lipid accumulation during adipocyte differentiation and re-expression of CD44 in CD44 KO cells abolished this effect, accentuating CD44-specific effects. Kang *et al*. reported that HFD-fed CD44 global KO mice had increased epididymal WAT mass with larger adipocytes and increased lipogenic gene expression (e.g. *Cidec, Fasn, Fabp1, Mogat2*) (Kang *et al*. 2013), indicative of enhanced adipogenesis and lipogenesis, which support our findings. Moreover, we provided additional mechanistic evidence how CD44 may regulate adipogenesis via modulating *Pparg* expression. Activation of *Pparg* by rosiglitazone has been previously shown to promote marrow mesenchymal stem cell U-33/*γ*2 differentiation with decreased CD44 (Shockley *et al*. 2007), suggesting a possible negative regulation between *Pparg* and CD44. Furthermore, our conditional culture medium studies confirmed that lack of *Pparg* activation significantly attenuated adipogenesis in both CD44KO EV and CD44KO RE cells but to a much greater extent in the CD44KO RE cells. Taken together, these results suggest that CD44 suppressed *Pparg* expression, therefore contributing to inhibited adipogenesis.

Deletion of CD44 in preadipocytes promoted lipid accumulation only after 7 days of differentiation initiation, which was lost by day 14. This suggests that CD44 may be more crucial in regulating mitotic proliferation and cell cycle arrest than terminal differentiation and maturation of adipocytes (Chang & Kim 2019). Indeed, our unbiased proteomics analysis and subsequent cell proliferation assay provided supporting evidence. The metascope pathway enrichment analysis of proteomics data suggests that cell cycle progression-related pathways, e.g. mitotic cell cycle process and mitotic cell phase transition, were most changed by deletion of CD44 in preadipocytes. Preadipocytes undergo unlimited proliferation until they become growth-arrested through contact inhibition (Lefterova & Lazar 2009). Under the stimulation of hormones such as insulin, the growth-arrested preadipocytes undergo cell cycle re-entry for mitotic clonal expansion and subsequent adipogenic induction (Lefterova & Lazar 2009). Interestingly, our proteomics data suggest that most proteins involved in all phases of the cell cycle were decreased by deletion of CD44 and was partially restored by re-expression of CD44. Furthermore, our cell proliferation assay showed that preadipocyte proliferation rate was increased by CD44 deletion and this increase was rescued by re-expression of CD44. While increased cell proliferation would contribute to increased adipogenesis in CD44KO cells, the discrepancy between changes in cell cycle genes and proliferation rates remains unclear. It is, however, possible that when the CD44KO cells were collected for proteomics analysis after 48 h of culture, they would have reached confluency due to enhanced proliferation rates; therefore, cell cycle arrest occurred with the concomitant reductions in cell cycle gene expressions. Regardless, this postulation merits further investigations.

One of the limitations of the current study is that 3T3-L1 cell adipogenesis was only assessed for 7 and 14 days. Our initial differentiation experiments (0, 7, and 14 days) suggest that deletion of CD44 primarily affects early differentiation (at day 7) but not at day 14, when the majority of 3T3-L1 preadipocytes are fully matured (Vishwanath *et al*. 2013). We then decided on day 14 as our terminal point to test the role of CD44 deletion on the function of mature adipocytes, minimizing the potential impact of differences in cell numbers of mature adipocytes. Thereafter, we kept day 14 as the terminal point for the re-expression experiments for consistency. It would have been interesting to explore the potential impact on the time course of differentiation. One other limitation of the study is that cell adipogenesis was assessed by Oil Red O staining. An investigation into the adipogenic potential of CD44KO and CD44 re-expressing cells in an *in vivo* setting would have been desirable. However, assessment of *in vivo* adipogenesis can be challenging. Tchoukalova *et al*. developed a method using the incorporation of the stable isotope deuterium (^2^H) into the DNA of isolated adipocytes and stroma-vascular fraction from adipose tissue to assess *in vivo* adipogenesis (Tchoukalova *et al*. 2012). However, this method was hampered by the potential contamination of adipocytes and their progenitors with other cell types. Alternatively, *ex vivo* adipogenesis can be assessed using adipose-derived stroma-vascular cultures. However, this approach also provides crude estimates of the adipogenic process because of the disruption of *in vivo* microenvironmental influences.

Blocking CD44 by genetic ablation or antibody antagonism attenuates adipose tissue inflammation, fibrosis, and insulin resistance with increased adiposity in obese mice (Kang *et al*. 2013, Kodama *et al*. 2015). Genetic deletion of CD44 also ameliorates HFD-induced skeletal muscle insulin resistance in obese mice (Hasib *et al*. 2019). CD44 abrogates insulin responsiveness upon activation by its ligands HA and OPN (Weng *et al*. 2022). Here, we found that re-expression of CD44 in CD44KO adipocytes decreased the insulin response by downregulating AKT phosphorylation (S473) and decreased adiponectin secretion. Adipose tissue stores excess energy in the form of triglycerides via *de novo* lipogenesis, adipogenesis, and adipocyte hypertrophy during obesity. Impaired adipogenesis pressurizes adipocytes into hypertrophy and subsequent adipocyte death, contributing to insulin resistance *in vivo* (Gustafson *et al*. 2015, Vishvanath & Gupta 2019). Thus, the regulation of CD44 on adipogenesis in preadipocytes may be crucial for determining insulin responsiveness and endocrine functions of mature adipocytes. Regardless, it is important to recognize that CD44 is primarily expressed in immune cells which were not tested in the current study but remains an important contributor to adipose function.

In conclusion, our study utilized CRISPR Cas9-mediated *Cd44* deletion and lentivirus-mediated *Cd44* re-expression techniques in 3T3-L1 cells and discovered that CD44 in preadipocytes is a critical regulator of adipocyte differentiation and the endocrine function of mature adipocytes. We are the first to study the direct role of (pre)adipocyte CD44 in adipose function, providing insight into a potential extracellular matrix-receptor regulatory component, the HA/OPN-CD44 signalling in adipocytes. It is currently unknown whether the favourably metabolic effects of CD44 deletion in obesity is through cell-autonomous actions or non-adipocytes, e.g. macrophages, and adipocytes interactions. Future studies using CD44 conditional KO mice to investigate cell type-specific role of CD44 in metabolism is essential. Nevertheless, our studies together with previous evidence suggest that CD44 may be a therapeutic target to treat obesity-associated metabolic diseases such as T2D.

## Declaration of interest

The authors declare that there is no conflict of interest that could be perceived as prejudicing the impartiality of the study reported.

## Funding

This work was supported by Diabetes UKhttp://dx.doi.org/10.13039/501100000361 (15/0005256 and 21/0006329 to LK) and British Heart Foundationhttp://dx.doi.org/10.13039/501100000274 (PG/18/56/33935 to LK). XW was supported by a PhD scholarship from China Scholarship Councilhttp://dx.doi.org/10.13039/501100004543.

## Author contribution statement

XW and LK contributed to the experimental design, researched data, contributed to discussion and data interpretation, and wrote the manuscript. HJ, DW, HZ, DL, and JW researched data, and reviewed and edited the manuscript.
